# Opportunities for *Helicobacter pylori* Eradication beyond Conventional Antibiotics

**DOI:** 10.3390/microorganisms12101986

**Published:** 2024-09-30

**Authors:** Camilia Metadea Aji Savitri, Kartika Afrida Fauzia, Ricky Indra Alfaray, Hafeza Aftab, Ari Fahrial Syam, Masrul Lubis, Yoshio Yamaoka, Muhammad Miftahussurur

**Affiliations:** 1 Department of Environmental and Preventive Medicine, Faculty of Medicine, Oita University, Yufu 879-5593, Oita, Japan; cmetadea@gmail.com (C.M.A.S.); rickyindraalfaray@gmail.com (R.I.A.); 2 Helicobacter Pylori and Microbiota Study Group, Institute of Tropical Disease, Universitas Airlangga, Surabaya 60286, Indonesia; kartikafauzia@gmail.com; 3 Research Centre for Preclinical and Clinical Medicine, National Research and Innovation Agency, Cibinong Science Center, Bogor 16915, Indonesia; 4 Department of Gastroenterology, Dhaka Medical College and Hospital, Dhaka 1000, Bangladesh; rosefebruary28@yahoo.com; 5 Division of Gastroenterology-Hepatology, Department of Internal Medicine, Faculty of Medicine, Universitas Indonesia, Jakarta 10430, Indonesia; ari_syam@hotmail.com; 6 Division of Gastroenterology-Hepatology, Department of Internal Medicine, Faculty of Medicine, Universitas Sumatera Utara, Medan 20155, Indonesia; masrullubisusu@gmail.com; 7 Department of Medicine, Gastroenterology and Hepatology Section, Baylor College of Medicine, Houston, TX 77030, USA; 8 Division of Genome-Wide Microbiology, Research Center for Global and Local Infectious Diseases (RCGLID), Oita University, Yufu 879-5593, Oita, Japan; 9 Division of Gastroentero-Hepatology, Department of Internal Medicine, Faculty of Medicine—Dr. Soetomo Teaching Hospital, Universitas Airlangga, Surabaya 60286, Indonesia

**Keywords:** antimicrobial resistance, *Helicobacter pylori*, eradication, biofilm, outer membrane vesicle, phage, human and health

## Abstract

*Helicobacter pylori* (*H. pylori*) is a bacterium known to be associated with a significant risk of gastric cancer in addition to chronic gastritis, peptic ulcer, and MALT lymphoma. Although only a small percentage of patients infected with *H. pylori* develop gastric cancer, Gastric cancer causes more than 750,000 deaths worldwide, with 90% of cases being caused by *H. pylori.* The eradication of this bacterium rests on multiple drug regimens as guided by various consensus. However, the efficacy of empirical therapy is decreasing due to antimicrobial resistance. In addition, biofilm formation complicates eradication. As the search for new antibiotics lags behind the bacterium’s ability to mutate, studies have been directed toward finding new anti-*H. pylori* agents while also optimizing current drug functions. Targeting biofilm, repurposing outer membrane vesicles that were initially a virulence factor of the bacteria, phage therapy, probiotics, and the construction of nanoparticles might be able to complement or even be alternatives for *H. pylori* treatment. This review aims to present reports on various compounds, either new or combined with current antibiotics, and their pathways to counteract *H. pylori* resistance.

## 1. Introduction

*Helicobacter pylori* is a Gram-negative bacterium known to infect half of the world’s population. In Asia, the prevalence of *H. pylori* infection exceeds 80% [1], while in Africa and Latin America, it is 79.1% and 63%, respectively [2]. Unless treated, the infection can persist for a lifetime [3]. It is mostly asymptomatic, but a small fraction of patients proceeds to develop gastric cancer. Inappropriate antibiotic treatment can lead to persistent infection, which favors progression to cancer [4]. More than 1 million new cases of gastric cancer are reported each year, causing more than 700,000 deaths in 2020 alone. Most patients with gastric cancer are diagnosed at an advanced stage, which worsens the prognosis [5]. Since 90% of gastric cancers are caused by *H. pylori* [2], the World Health Organization (WHO) has concluded that this bacterium is a class I carcinogen [6].

Due to the global prevalence, experts have attempted to formulate appropriate guidelines for managing *H. pylori* infection. Some of these guidelines include the Maastricht VI/Florence consensus, the Toronto consensus, and the American College of Gastroenterology guidelines [7,8]. First-line treatment for *H. pylori* infection consists of a proton pump inhibitor (PPI) and three or four penicillin-based antibiotics or fluoroquinolones with macrolides and metronidazole [1]. Clarithromycin triple therapy is the first choice when the resistance rate is below 15% [7]. Bismuth quadruple therapy is preferred in cases of high clarithromycin resistance [9]. Sequential therapy with PPI and amoxicillin for 5 to 7 days, followed by PPI, clarithromycin, and nitroimidazoles for the next 5 to 7 days, has also been proposed as an alternative treatment for clarithromycin resistance [6]. However, the WHO has recognized clarithromycin-resistant strains as one of the priority pathogens needing new antibiotics [10].

As antimicrobial resistance evolves, treatment failure increases [11]. Resistance to clarithromycin reduces the success rate of triple therapy by up to 66%, while resistance to metronidazole reduces the success rate by up to 38%. In addition, MDR strains of *H. pylori* have reached 40% in some regions of the world, threatening the eradication of this bacterium [12]. MDR strains arise from antibiotic consumption patterns, comorbidities, bacterial characteristics, and patient compliance [2,9,13,14]. However, there is a slight discrepancy between the genotypic characteristics and the phenotype of the bacteria, raising the question of whether a different mechanism is used to confer resistance [15,16,17,18,19,20]. Since the efficacy of conventional antibiotics is reduced when there is a mutation on the drug targets, research has been conducted to find proper strategies to combat resistance. Our understanding of bacterial survival mechanisms has progressed over the years, which can be exploited to produce narrow-spectrum antimicrobials. This review focuses on advances in alternative eradication methods for *H. pylori*, ranging from the use of bacterial parts and the inhibition of bacterial pathogenesis to bioengineered drug carriers.

## 2. Opportunities beyond Traditional Antibiotics

Antimicrobial resistance is a challenge in the management of infectious diseases. Various approaches have been taken to address this problem. In addition to optimizing the efficacy of existing antimicrobials through new drug carriers and finding new potential substances with eradication ability, *H. pylori* pathogenesis has been exploited as a drug target, and its bacterial part has been used for therapeutic purposes.

### 2.1. Eradicating Biofilm

Bacteria living in hostile conditions form biofilms to protect themselves [21]. Biofilms are related to chronic infectious diseases due to their additional protection against environmental and host factors [3]. Stress conditions, such as starvation, oxidative stress, and subinhibitory antibiotic concentrations, could trigger biofilm formation [22]. They attach using an extracellular polymeric substance (EPS) that contains polysaccharides, proteins, and extracellular DNA (eDNA). Bacteria adhere to a surface and then multiply and aggregate with EPS, leading to a microcolony. EPS stabilizes the 3D structure of the biofilm and protects bacteria from various stresses [23]. The bacteria tightly attach to the surfaces, causing corrosion, food contamination, and infection due to their attachment to medical devices [21]. Moreover, biofilm formation can increase antibiotic resistance due to poor drug penetration, a slow growth rate, and the adaptive stress response triggered by the bacteria [24]. A slow growth rate results in decreased antibiotic efficacy, which requires active and growing cells to function properly [25]. The biofilm environment supports the spread of antibiotic resistance genes through horizontal gene transfer, integrative conjugative elements, and natural transformation [3].

*H. pylori* is capable of biofilm formation in vitro and in vivo [21,26]. Biofilm formation is controlled by quorum sensing (QS) signaling molecules, such as autoinducing peptide (AIP), autoinducer-2 (AI-2), N-acyl-homoserine lactones (AHL), and diffusion signaling factor (DSF). In addition, mutations in various genes were associated with the hyperbiofilm phenotype. Although the mechanism of *H. pylori* biofilm resistance is not fully understood, the involvement of the EPS and efflux pump is believed to contribute to the resistance. The EPS encapsulates the bacteria, thereby reducing antibiotic penetration and preventing the host immune system from directly interacting with the bacteria [23]. *H. pylori* neutrophil-activating protein A (NapA), primarily used to protect DNA from iron-mediated oxidative stress, is involved in forming the extracellular matrix. The extracellular matrix protects *H. pylori* from reactive oxygen species (ROS)-mediated toxicity [22]. Moreover, antimicrobials lose their inhibitory and bactericidal activities for dormant bacteria within the biofilm [23]. As for efflux pumps, studies showed a higher expression of efflux pump genes when cells formed biofilm [4,26].

One study showed that the minimal biofilm eradication concentration (MBEC) was higher than the minimal bactericidal or inhibitory concentration (MBC or MIC) [27]. In the *H. pylori* strain TK1402, biofilm formation increased the minimum bactericidal concentration by up to 8-fold for amoxicillin and 2-fold for metronidazole. In contrast, for strain TK 1049, the values were increased by 4-fold and 2-fold, respectively [21]. A study of Indonesian strains showed a significant difference between the MBEC and MIC. The MBEC for amoxicillin was increased by 1000-fold, while a 31-fold increase was observed for tetracycline and clarithromycin [28]. Another study of clarithromycin resistance showed that biofilm formation in *H. pylori* increased the MIC levels by up to 2-fold in 2-day biofilm, while a more striking 16-fold result was observed in 3-day biofilm [26]. A study comparing the susceptible, mono-, bi-, and multi-drug resistant strains showed strong correlations between clarithromycin resistance and biofilm formation, as all strains with clarithromycin resistance are strong biofilm formers [29].

Biofilm formation is difficult to predict by routine antimicrobial resistance testing [28]. Therefore, it is more complicated to eradicate *H. pylori* without knowing whether biofilm formation will occur. N-acetylcysteine (NAC), commonly used as an antioxidant, can disrupt mucin disulfide bonds and act as a mucolytic agent. In the gastric environment, NAC can reduce mucus thickness, inhibit biofilm formation, and eradicate premature biofilm [30]. Despite being the only molecule effective against *H. pylori* in clinical trials [23], a meta-analysis showed that combining NAC with standard *H. pylori* eradication therapy was not superior to the standard treatment alone [31].

**Figure 1:**
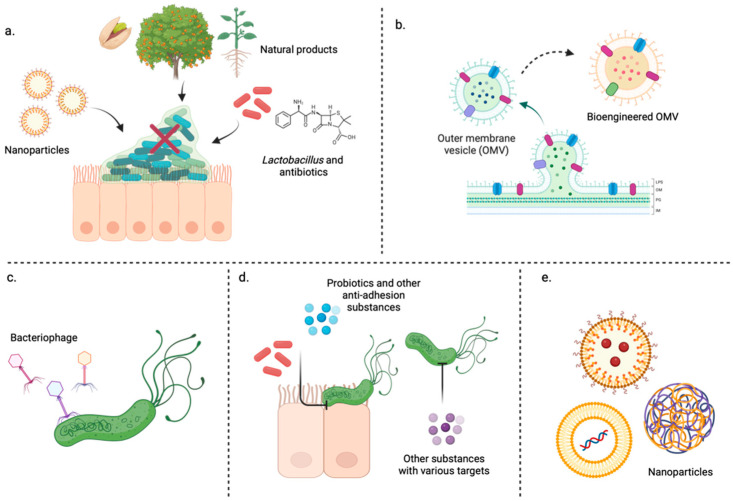
Alternatives to *H. pylori* eradication. (**a**) Biofilm formation protects bacteria from antimicrobials. Multiple natural products have anti-biofilm activity. Modifying the drug carrier with nanoparticles allowed the drug to pass through the biofilm and kill the bacteria. The combination of probiotics and conventional antibiotics could help to destroy biofilms. (**b**) OMVs in *H. pylori* can protect the bacteria under various stress conditions. However, these OMVs can be modified as natural drug carriers and vaccines. (**c**) Bacteriophages are natural predators of bacteria. (**d**) The utilization of bacterial pathogenesis via anti-adhesion or molecular processes inhibiting substances. (**e**) The construction of nanoparticles containing various cargos ranging from antibiotics to nucleic acids. (Made with biorender.com, accessed on 14 September 2024).

Several antibiofilm agents are derived from natural products (Figure 1a). Some, such as *Pistacia vera*, dihydrotanshinone I, and armeniaspirol A, are even effective in vitro and in vivo [23]. *Pistacia vera* worked synergistically with levofloxacin [32], while a combination of armeniaspirol A and omeprazole was more effective in eradicating *H. pylori* in an MDR mouse model [23]. Plant materials from various *Rubus* genera of their fruits and shoots prevent biofilm formation. The shoot extract from *Rubus idaeus* “Willamette” synergized with doxycycline and levofloxacin [33]. Citropten, one of the coumarins extracted from *Citrus sinensis* leaf, completely inhibits *H. pylori* planktonic growth by binding to one of the *H. pylori* enzymes [34]. The ethyl acetate fraction of *Hibiscus rosa sinensis* and phylligenin isolated from *Forsythia* also exhibit anti-biofilm activity [35], while eugenol essential oil extracted from cloves exhibits both anti-biofilm and anti-inflammatory activities [36]. A phylligenin derivative can inhibit *H. pylori* colonization and biofilm formation in vivo [37]. Other spices, turmeric, had curcumin, which inhibited *H. pylori* urease, growth, and biofilms in the dose and strain-dependent manner [38]. In addition, aloe-emodin from *Aloe vera* targets the *H. pylori* outer membrane protein-6 (OMP6) and disrupts biofilms [39]. An artemisinin derivative showed an inhibitory effect on *H. pylori* biofilm formation [40]. Plants used in traditional medicine to treat disease, such as *Astractylodes lancea* [41] and *Acorus calamus* [42], can also inhibit biofilm formation. Another common traditional Chinese medicine, *Cinnamomum cassia*, inhibits both sensitive and resistant strains. The combination of PPI and *C. cassia* inhibits adhesion, colonization, and biofilm formation in vivo [43], while dihydrotanshinone I (DHT) derived from the dried root and rhizome of *Salvia miltiorrhiza* killed biofilm-encased *H. pylori* better than metronidazole. In vivo, dual therapy of DHT and omeprazole showed superior efficacy than standard triple therapy [44]. Myricetin, isolated from the bark of the Myrica tree, can slow down *H. pylori*’s transformation into coccoid and then reduce biofilm [45]. Other plant extracts include *Acacia nilotica* flower extract [46], laurel leaf extract [47], *Chelidonium majus*, and *Corydalis cheilanthifolia* [48]. Moreover, quinones, which are widely found in plants and animals, can kill *H. pylori.* One of the synthesized quinones, called M5N32, had an inhibition effect on both planktonic and biofilm states in vitro [49]. Although many plant extracts have anti-biofilm effects, the bioactive components need further investigation. Moreover, environmental factors can cause variations in plant compositions, changing the content of probable bioactive, affecting the drug performance. The plant’s age, harvesting process, and extraction parameters must also be standardized. Balancing with the patient number, the quantity of the final product needs to be calculated, which probably leads to over-exploration in the environment [50]. As for probiotics, combining *Lactobacillus salivarius* with amoxicillin and clarithromycin destroyed the biofilm structure in vivo, thereby enhancing the therapeutic effect of antimicrobials [51]. At the same time, combining *Lactobacillus* with levofloxacin could improve the eradication effect of *H. pylori* biofilm [52]. *Bacillus* sp. 1630F and *Enterococcus* sp. 7C37 could form biofilm and combat *H. pylori* biofilm formation [53]. In addition, some antimicrobial peptides (AMP), such as pexiganan, IDR-1018, and DJK-5, had antibiofilm activity [54]. A summary of the anti-biofilm alternatives can be found in Supplementary Table S1.

Since biofilm is naturally hard to penetrate, drug modification strategies such as nanoparticles are used [55,56]. A self-assembled nanodrug containing berberine derivatives and rhamnolipids can penetrate the mucus, destroy EPS, and kill planktonic *H. pylori* both in vitro and in vivo [57]. Grande et al. generated a silver ultra-nanocluster (SUNCs) that shows a synergistic effect in combination with clarithromycin and metronidazole. It could eradicate *H. pylori* and disrupt the biofilm and has low toxicity [58]. A lipid polymer nanoparticle with encapsulated clarithromycin significantly reduces the biofilm biomass and viability. It penetrates the mucus layer without interacting with mucins [59]. A nanoparticle blend with rhamnolipid, cholesterol-PEG, and calcitriol encapsulating clarithromycin destroys biofilms and inhibits bacterial re-adhesion [60]. An innovation that can identify and evaluate the susceptibility and pharmacokinetics of six anti-biofilm drugs is made based on AlpB, an outer membrane protein involved in biofilm formation. This indicates that the tested drugs can destroy *H. pylori* biofilm by acting on AlpB [61]. Although most studies are performed in vitro, a promising preliminary result offers hope for eradicating biofilm and combating antimicrobial resistance.

### 2.2. Outer Membrane Vesicles (OMVs) and Their Versatility

*H. pylori* releases outer membrane vesicles (OMVs) during its growth [62]. OMVs are small, approximately 20 to 500 nm in diameter, and contain phospholipids, proteins, lipopolysaccharides, DNA, and RNA. They are part of the biofilm matrix in various Gram-negative bacteria [3]. OMVs have been implicated in bacterial survival, host–bacteria interactions, toxin and virulence factor delivery, and gene transfer [63]. In *H. pylori*, OMVs are released into the extracellular space [64]. They contain virulence factors such as CagA [65] and VacA [66]. OMVs were observed in the strong biofilm-forming strain, TK1402, but not in the low biofilm-forming strain [3,67]. Moreover, the strong biofilm-forming strain showed larger OMVs (50 to 300 nm) than the weak biofilm-forming strain (50–80 nm) [29]. In addition to biofilm, the role of OMVs in host-pathogen interactions has also been studied [68].

In the other bacteria, OMVs can protect against antibiotic exposure. OMVs from *Escherichia coli* can protect bacteria against membrane-active antibiotics [69]. Moreover, OMVs derived from β-lactam-resistant *E. coli* could rescue susceptible *E. coli* among other bacterial species [70]. *Staphylococcus aureus* was protected from β-lactam antibiotics by β-lactamase’s presence in the vesicles [71]. However, studies investigating the protective effect of OMV in *H. pylori* are limited. A study showed that OMVs protect *H. pylori* against clarithromycin in a dose-dependent manner. This phenomenon is probably due to OMVs acting as a decoy for clarithromycin diffusion. OMV supplementation also allows *H. pylori* to grow despite the presence of antimicrobial peptide LL-37 [62]. OMVs released from *H. pylori* exposed to bismuth had accumulated bismuth to the point that it was undetectable in the bacterial cell. This showed that *H. pylori* can use OMVs to cope with antimicrobial stress [72]. In addition, OMVs can protect *H. pylori* under oxidative stress [73]. OMVs in other bacteria can transfer resistance genes because they carry DNA and RNA [74,75]. However, OMVs have not been shown to transfer genes in *H. pylori*.

Research on the prevention of OMV release is scarce. Two plant metabolites, carvacrol, and thymol, are selective *H. pylori* carbonic anhydrase inhibitors that could inhibit OMV release and biofilm formation [76]. Despite the role of OMVs as a virulence factor, the therapeutic potential of bacterial membrane vesicles has recently been under investigation. The structure of an OMV is promising for vaccine development because it can carry surface antigens and be engineered to carry therapeutic agents. It rapidly enters the tissues and interacts with various parts of the immune system [74]. In *H. pylori*, OMVs induce apoptosis in Jurkat T cells and naïve CD4+ cells [77]. The oral administration of *H. pylori*’s OMVs to mice showed that host NOD1 recognizes the peptidoglycan and induces innate and adaptive immunity in the mucosal compartment [78]. A similar result was displayed by Liu et al., who tested the immune response of *H. pylori*’s OMVs in a mouse model, generating strong mucosal and humoral responses without inflammation that reduced the *H. pylori* load [79]. While two OMV-based vaccines are currently available for *Neisseria meningitidis* [80], there is currently no OMV-based vaccine for *H. pylori.* However, its OMVs have been studied as vaccine adjuvants for the outer membrane protein (OMP) and whole-cell vaccines (WCVs) [81]. Adjuvants are used to enhance antigen-specific immune responses [74]. Commonly used adjuvants for *H. pylori* vaccination are cholera toxin (CT) and *E. coli* heat-labile enterotoxin (LT). However, the *H. pylori* OMV adjuvant has shown safety and efficacy advantages by enhancing humoral and innate immunity via Th2- and Th17 [81]. Therapeutic OMVs could be prepared from different bacteria to treat various infectious diseases. Bioengineered OMVs have higher biocompatibility by reducing toxicity, combining heterologous antigens, and selectively attaching target molecules to act against bacterial infection [82]. Liu et al. constructed an OMV from *Salmonella* mutants with *H. pylori*’s UreB, VacA, and CagA. OMVs from ∆*rfb* ∆*fliC* ∆*fliB* ∆*ompA* from *S. typhimurium* as a vector for UreB and CagA can protect against *H. pylori* in mice. These findings might increase the chances of developing a vaccine for this bacteria [83]. The development of an *H. pylori* vaccine is in its early stage, with only one trial in phase 3 [84]. The versatility of OMVs as an adjuvant or delivery system opens the opportunity to expand vaccine candidates (Figure 1b).

OMVs have been used as coatings for nanoparticles for their immunogenicity and antibacterial ability. *H. pylori* OMV-coated nanoparticles can reduce bacterial adhesion by competing for binding sites [82]. As natural drug carriers, OMVs can easily fuse with the membrane of the target bacteria and have a prolonged circulation time. OMVs with appropriate stability and biocompatibility can be prepared from available parent bacteria [85].

Despite their versatility, the use of OMVs is fraught with challenges. Several issues related to the use of bacterial OMVs include LPS toxicity, leading to sepsis [86], bacterial viability, appropriate culture conditions, OMV isolation, and purification methods that affect cargo selection and the choice of proper presenting antigen [85,87]. Attenuating toxicity through the extraction of toxic components or genetic modification of the bacteria are some of the methods researchers use to increase the safety of OMVs. In Gram-negative bacteria, OMVs may carry virulence factors that must be considered before using OMVs [88]. Therefore, developing methods to induce OMV production appropriately and adequately and bioengineering to prevent potential toxicity and side effects to ensure safety are necessary to use *H. pylori*’s OMVs as therapeutic agents.

### 2.3. Phage Therapy

A mobile genetic element (MGE) can be defined as any genetic element that can be transferred by horizontal or vertical gene transfer between bacterial chromosomes within or between species [89,90,91,92]. *H. pylori* has been observed to acquire MGE genes by natural transformation, both in mouse infection and in colonized human models [93,94,95]. Several MGEs, including plasmids, phages, and insertion sequences, have been reported in *H. pylori* [96,97,98]. Plasmids and phages can possess and spread antibiotic resistance genes, while smaller MGEs, such as the insertion sequence (IS), can affect gene expression before or after the integration sites [99,100].

One of the new alternative treatments for antimicrobial-resistant strains is phage therapy using bacteriophages. A phage is a bacterial virus, a natural predator of bacteria. Hence, it has host specificity and a narrow spectrum activity, refraining from disrupting the gut microbiota [101,102]. Bacteriophages are easy to isolate, replicate only in the target bacterium, encode enzymes to overcome biofilm formation, can be personalized or combined as cocktails, coevolve with the host to adapt to resistant bacterial strains, are potentially stable in vivo, and are effective in treating various infections [97,103]. They follow one of two infection cycles: lytic or lysogenic (temperate) [104]. Lytic phages inject nucleic acid into the bacterial cell, replicate, and then release new phages to infect other bacteria. Lysogenic phage DNA integrates its genome into bacteria, forming a prophage capable of horizontal gene transfer [97,105].

Phage therapy primarily uses lytic phages to kill bacterial hosts while sparing human cells. Its use dates back to 1917 for the treatment of severe dysentery [103]. In vivo studies have shown a beneficial effect of phages against *A. baumanii*, *P. aeruginosa*, *E. coli*, and *K. pneumonia* [106]. The use of phages has been a therapeutic modality in Eastern Europe and the former Soviet Union [103,106]. It is used on a case-by-case basis [105]. In some cases, phages have been used to treat biofilm infections safely and effectively [107]. However, studies using a phage as *H. pylori* therapy are scarce, as unavailable genomic analyses limit these studies [97]. In addition, the harsh environment of the stomach could alter the structural and biological properties of the phage. Using a coating that protects the phage could be a solution for this.

*H. pylori* phages have been discovered over the years [108,109,110,111,112]. Hpφ, a lytic bacteriophage, showed antimicrobial activity in vitro. It was constructed as a combination of phage–lactoferrin–hydroxyapatite and exerts antimicrobial activity that is four times greater [101]. Due to the difficulties in isolating *H. pylori* lytic phages, attempts were directed toward isolating prophages. The most recent phage studied was HPy1r, isolated from a clinical strain and induced by UV light. This phage showed stability under gastric conditions and inhibited *H. pylori* in vitro for up to 24 h post-infection at different multiplicities of infection (MOIs) [104]. *H. pylori* had an impenetrable lipopolysaccharide that prevented endolysin, which is used by phages to facilitate host–cell lysis, from interacting with the cell wall. Xu et al. engineered an artilysin that linked an endolysin enzyme to holin with polypeptides that perforated the surface of *H. pylori* and destroyed it [113]. Thus, using a phage may also be an alternative for eradicating *H. pylori* (Figure 1c).

However, a phage is a self-replicating virus, so dosage is a concern. The massive lysis of bacteria potentially releases antigens that can be dangerous [105]. A phage also requires direct contact with the bacteria to exert its effect. Because of the precise mechanism of action, multiple bacterial infections will likely require phage cocktails or combination therapy with antibiotics [106]. In addition, the emergence of phage-resistant bacteria is inevitable. This adaptive mechanism could be advantageous if the mutation to resist the phage reduces the bacterial defense against antibiotics. Bacteria could modify phage receptors, use OMVs to avoid phages, use quorum sensing, or use a restriction–modification system and a CRISPR/Cas system. These phenomena have been demonstrated in the MDR *P. aeruginosa* clinical isolate exposed to virulent phage OMKO1, causing the bacteria to lose its efflux pump-associated outer membrane protein and regain its resistance to antibiotics [114]. There are currently no studies of phage-resistant *H. pylori.* Establishing phage banks and ensuring phage safety, stability, and quality during production are concerns. A dynamic biophysical condition (pH, digestive enzymes, and transit time) negatively impacts the viability and potency of orally administered phages [115,116]. The complexity of the pharmacokinetics and pharmacodynamics of phage therapy is increased when phages are used in combinations. Researchers tried to overcome these by engineering phages, modifying their delivery systems by using nanoparticles, using phage components instead of a whole phage, making phage cocktails, and combining phage therapy with other antimicrobial agents [114,117].

There are only a few phage therapy centers worldwide, with varying ethics and regulations [116]. The Belgian authorities stated the need to issue a written form of the quality of a phage active pharmaceutical ingredient (API), which certified that phage stock is prepared by a pharmacist based on a prescription from a physician. Similar regulations are also imposed in Georgia and Russia [118]. Eventually, more research will be needed to standardize and improve the safety and efficacy of phages and pave the way for regulations on using phages for *H. pylori* eradication.

## 3. Various Ways to Target *H. pylori*

Efforts have been made to develop narrow-spectrum antibiotics, such as compounds targeting menaquinone synthesis, which is essential for *H. pylori*’s survival [119], compounds targeting respiratory complex I, which involves an electron transport chain [120], or for targeting flavodoxin, whose inhibition blocks electron transfer necessary for cell survival [121]. Some natural products, such as docosahexaenoic acid (DHA), a polyunsaturated fatty acid (PUFA) that can cause bacterial lysis, have shown a significant reduction in *H. pylori* growth both in vivo and in vitro [122]. Bacteria inside the cells are protected from eradication attempts. Therefore, the activation of the cellular autophagy pathway in the host cell could eradicate the bacteria. Vitamin D3 treatment activates lysosomal function in the normal gastric epithelial cell [123]. In addition, an agonist of transient receptor potential membrane channel mucolipin 1 (TRPML1), an endo lysosomal calcium channel, could eliminate the intracellular *H. pylori* reservoir [124]. An anti-motility compound of phenyl-pyrazolone small compound Active2, termed antimotilin, is capable of reducing the bacterial load in mouse models without altering the microbial composition; an alteration warrants further investigation in *H. pylori* pathogenesis blockers [125]. *Lactobacillus gasseri* inhibits *H. pylori*’s motility, probably through the downregulation of *flgR* [126]. Moenomycin, which inhibits trans glycosylase to block peptidoglycan formation, can eradicate *H. pylori* in vitro. Its efficacy is increased in combination with clarithromycin, even against MDR strains [127].

Since adhesion is crucial in *H. pylori* pathogenesis, anti-adhesion therapy has been proposed to control the bacteria. Various sialic acid-based compounds and sialic acid-based delivery systems have been identified and developed, as they can effectively inhibit bacterial adhesion and enhance eradication [6]. Polysaccharide sulfates could inhibit *H. pylori*’s adhesion to red blood cells and AGS cells through competitively binding to *H. pylori.* As a result, *H. pylori* could not bind to the host [128]. Henriques et al. constructed a chitosan microparticle, a natural polysaccharide with mucoadhesive properties, to bind to *H. pylori* and clear the bacteria from the gastrointestinal systems of mice [129]. Dextran sulfate, a polysaccharide formulated in nanocapsules, could block *H. pylori* interaction with MKN45 cells. It dissociates *H. pylori*–mucin complexes and inhibits binding to mucins at an acidic pH [130]. Plant-derived substances have also been evaluated for their anti-adhesive ability toward *H. pylori* [131,132].

Probiotics are another alternative gaining interest as an adjunct to standard antibiotics due to their ability to produce antibacterial substances, regulate the immune system, inhibit adherence competition, maintain intestinal epithelial homeostasis, etc. [133]. A screening of probiotic candidates showed that *Lactobacillus gasseri* and *L. rhamnosus* induced a decrease in the hummingbird appearance in AGS cells infected by *H. pylori.* Both strains inhibit epithelial-to-mesenchymal transition (EMT) and the pro-inflammatory response triggered by *H. pylori* [134]. *Lactobacillus* spp. and even their supernatant showed an inhibitory growth effect and inhibited adherence in *H. pylori* [135]. *L. casei*, *L. paracasei*, and *L. acidophilus* can inhibit fifty-seven *H. pylori* strains [136], while *L. fermentum* UCO-979C inhibits *H. pylori* strain SS1 in Mongolian gerbils [137]. *L. fermentum P2* (P2), *L. casei L21* (L21), *L. rhamnosus JB3* (JB3) reduce gastric inflammation in mice [138]. One way probiotics compete with *H. pylori* for adherence is through lactic acid production, as demonstrated by *L. salivarius*, *L. acidophilus*, *L. rhamnosus*, and *L. casei* [133,135]. *L. plantarum* competes for *H. pylori* binding sites, resulting in decreased TNF-α expression and increased IL-10 [139]. In vivo, *L. plantarum* ZFM4 reduces gastric inflammation caused by *H. pylori* [140]. *L. casei* downregulates TNF-α and MUC5AC, IL-6, and IL-8 expression in vitro [141]. *Saccharomyces boulardii*, a yeast, prevents *H. pylori* adhesion and exerts an immune-protective effect with immunoglobulin secretion [142].

Probiotics can facilitate the inhibition of *H. pylori* growth by antibacterial substances (Figure 1d). They secrete short-chain fatty acids (SCFAs), lactic acids, hydrogen peroxide, and bacteriocins. Lactic acid lowers pH, inhibiting *H. pylori* urease activity, while bacteriocins produced by various *Lactobacillus* have antagonistic activity against biofilms [143]. Bacteriocins inhibit growth by forming pores in the membrane, activating autolytic enzymes, and downregulating the expression of *vacA*, *cagA*, *luxS*, and *flaA* genes. Nevertheless, bacteriocins are strain-specific and sensitive to enzymes in the gastrointestinal tract [144]. *L. reuteri* secretes reuterin, which can inhibit *H. pylori* growth and reduce the product of the *VacA* gene [145]. *Lactobacillus* can reduce the adverse effects of antibiotic therapy, especially when given for more than two weeks [8]. In addition, probiotics can strengthen the gastric mucosal barrier by altering the expression of mucus and epithelial junction proteins and releasing barrier-stabilizing molecules [146,147].

The clinical application of probiotics in *H. pylori* treatment has been performed in many countries. A meta-analysis of 8924 patients concluded that combining probiotics with a bismuth quadruple regimen improves the eradication rate (relative risk (RR) 1.14; 95%CI: 1.10–1.18; *p* < 0.001) and reduces side effects (RR 0.47; 95%CI: 0.39–0.57; *p* < 0.001), with *Lactobacillus* and multiple strains producing better eradication effects [148]. On the other hand, no significant difference in eradication rates was observed with the combination of bismuth quadruple therapy (BQT) and *Clostridium butyricum* probiotics [149], BQT with *Bacillus subtilis* and *Enterococcus faecium* [150], and BQT with four different species, namely *Bifidobacterium infantis*, *L. acidophilus*, *E. faecalis*, and *B. cereus* [151]. In addition, when administered alone, the eradication rates are still low [152,153]. An RCT in Greece using a 10-day proton pump inhibitor (PPI) and non-bismuth quadruple therapy resulted in increased eradication rates with fewer side effects compared to a placebo [154]. Various probiotics are probably involved in restoring gut mucosal homeostasis, while some only result in fewer changes [148]. Patients with probiotics have fewer fluctuations in gastric microbiota, which probably contributes to the fewer adverse effects observed [151]. Moreover, probiotics can modulate the host immune system, as evidenced by lower levels of TNF-α, IL-6, and IL-8 in patients given *L. plantarum* Q21, Q25, and QA85 [155]. Probiotics are not routinely used due to the uncertainties regarding a combination of strains, dosages, durations of therapy, and side effects. Moreover, probiotic products’ formulations, quality, production, and shelf life need to be considered.

## 4. New Drug Delivery Systems

Nanoparticles (NPs), novel drug delivery systems, are widely used in antimicrobial therapy. They are small, ranging from 1 to 1000 nm. Nanoparticles include lipid nanoparticles, liposomes, polymeric micelles, metallic NPs, and polymeric NPs [156]. Various nanoparticle compositions have been developed that encapsulate either antibiotics, or other antibacterial substances have been created. Liposomes are spherical, chemically similar to cell membranes, and have a low frequency of allergic reactions and systemic toxicity through ingestion. When injected, it is difficult for the reticuloendothelial system to absorb these vesicles. Hence, various coatings are used to escape the immune system [157]. One of the most popular applications of lipid-based particles is two vaccines against COVID-19: Moderna and Pfizer. It provides a safe and stable structure that can carry mRNA or other components [158]. Lipid-based nanoparticles can kill *H. pylori* inside the cells and biofilms (Figure 1e). Despite having a complex construction process, it can protect the contained drugs from gastric acidity, increase membrane permeability, and alter the membrane structure of bacteria [159]. In addition, it can persist longer in the stomach, allowing prolonged contact between the bacteria and the drug, which optimizes mucosal penetration and adherence to the bacteria [160]. A pectin-coated liposome could induce mucus adhesion, penetrate it, adhere to *H. pylori,* and release its amoxicillin content [161]. Metallic NPs can easily penetrate the peptidoglycan cell wall of bacteria, inhibit oxidative phosphorylation, and alter DNA replication. They contain either silver, zinc, or bismuth. While silver NPs can reduce biofilm formation in vivo, functionalized zinc oxide NPs can inhibit metronidazole-resistant *H. pylori,* causing severe membrane damage in the bacteria. They can be used in combination with conventional antibiotics [156]. One example of polymeric NPs, ethyl cellulose nanoparticles with encapsulated clarithromycin, had anti-adhesion activity in vivo and eradicated *H. pylori* better than non-capsulated clarithromycin in vivo. DHA encapsulated in a nanostructured lipid carrier has higher bactericidal efficacy than DHA alone [162]. A polymeric nanoparticle coated with *H. pylori*’s OMVs (OM-NPs) could compete with the bacteria for cell surface binding sites [163]. NPs coated with the plasma membrane of AGS cells loaded with clarithromycin showed better therapeutic efficacy than the free drug [164]. In addition, nanoparticles could be designed to target *H. pylori* specifically. A ureido-conjugated chitosan derivative nanoparticle called UCCs-2/TPP that specifically targets the urea transport channel, UreI, combined with polylactic-co-glycolic acid (PLGA) to improve biocompatibility, was constructed. Amoxicillin was loaded inside. The results show that this nanoparticle is pH-adapted to gastric acid and has a superior anti-*H. pylori* effect than regular amoxicillin [165]. A pH-responsive metal-organic framework hydrogen-generation nanoparticle kills *H. pylori*, alleviates inflammation, and restores impaired gastric mucosa (10.1002/adma.202105738). In addition, a sonodynamic therapy mediated by lecithin bilayer-coated poly nanoparticle loaded with verteporfin inactivates *H. pylori* by generating ROS [166].

Another compound under investigation is a synthesized nanozyme capable of catalyzing reactive oxygen species (ROS) production under acidic conditions and specifically targeting *H. pylori* to generate ROS only around the bacterial membrane [167]. Furthermore, nucleic acid mimics (NAMs) have emerged as an alternative to overcome *H. pylori*’s antimicrobial resistance. Locked nucleic acid (LNA) and 2′-Omethyl RNA (2′OMe) oligonucleotides could be hybridized into *H. pylori* and act as an antisense therapy by inhibiting mRNA translation [168]. PEGylated liposomes are designed to facilitate the delivery of NAMs across mucus and into *H. pylori*. However, the delivery efficiency is reduced due to the interaction of liposomes with the outer membrane of bacteria [169]. The size and shape of NP depend on its content, while its stability depends on temperature. Hence, uniform and stable engineering NPs are necessary to utilize this carrier [170]. Most clinical trials on nanoparticles involved chemotherapy drugs. On the other hand, pain management drugs had the most trials in phase 4 [171]. Various nanomedicines had been approved and used in clinical settings; most were for cancer, and very few were for infectious diseases [171,172].

Despite the attractive use of new drug delivery systems to improve the eradication of *H. pylori*, the use of nanoparticles is shadowed by toxicity. Toxicity can be mediated by an inflammatory response, producing reactive oxygen species and causing damage to the body [172,173]. Nanoparticles can accumulate in lymphoid organs or kidneys, leading to overexposure [174]. For polymeric nanoparticles, non-specific interactions can occur with cells or opsonizing proteins in the bloodstream, which may cause unexpected toxicity. Silver nanoparticles cause in vitro toxicity and DNA damage in human cell lines [175]. Standard toxicology tests also did not account for complex human physiology, and the traceability of nanomaterials needs to be considered [172]. People who work with nanomaterials are also at risk of accidental exposure via inhalation or dermal contact, which can cause severe toxicity. Moreover, these nanoparticles can be released into the environment [176], either during production, after use, or upon disposal [172]. Changes in raw materials and manufacturing processes affect nanomaterials’ characteristics. Hence, controlling critical points during manufacture is necessary, especially regarding the reproducibility of nanomaterials in large-scale production. In addition, the cost of materials and production process makes nanomedicine very expensive [177]. To be used in the clinical setting, the potential for improved patient benefit must be considered. Benefits can include increased efficacy, less toxicity, less frequent dosing, or a more convenient administration route. However, when the existing drug is cheap and can be taken orally, physicians and patients might think twice before changing to nanomedicines with the parenteral route [174]. Although some nanomedicines have been approved, there are no general protocols and specific guidelines for them. Some countries have tried to build entities to regulate this field better [175], such as the Nanotechnology Task Force by the Food and Drug Administration (FDA) and the Health Portfolio Nanotechnology Working Group in Canada [172]. As the interaction of nanoparticles and the human body is not fully understood, further research with proper guidance on physicochemical properties, administration routes, dosages, and toxicities are needed to advance the field.

## 5. Conclusions

Intensive research has been conducted to find alternatives to combat antimicrobial resistance in *H. pylori*. Since antibiotic research has taken longer than the ability of bacteria to gain resistance, efforts are being made to maximize the use of antibiotics by constructing nanoparticles as drug delivery systems that allow the drug to persist longer in the bloodstream and easily penetrate the bacteria. In addition, OMVs are being used as natural carriers or as vaccine adjuvants. Interfering with the bacteria’s ability to survive in its adhesion, motility, and molecular processes and biofilm formation is another avenue being explored to eradicate these bacteria despite the frequent mutations in drug targets that cause resistance. Phages, a natural bacterial predator, are being sought in the obscure phage field of *H. pylori* with the hope of using them in phage therapy. However, there are challenges in the production, pharmacokinetics, pharmacodynamics, safety, and stability of these new modalities. Moreover, regulations and distributions of these new modalities need to be implemented to ensure the benefit applies to the population.

## Data Availability

The data are available under the corresponding authors’ authority.

## Supplementary Materials

The following supporting information can be downloaded at: https://www.mdpi.com/article/10.3390/microorganisms12101986/s1, Table S1: Summaries on *H. pylori* eradication alternatives.
