# Supplementary material for: Opportunities for Helicobacter pylori Eradication beyond Conventional Antibiotics
Source: Microorganisms. 2024 Sep 30;12(10):1986. doi: 10.3390/microorganisms12101986 (PMC11509656; doi:10.3390/microorganisms12101986)
Supplement: Supplementary file 1 [file microorganisms-12-01986-s001.zip › microorganisms-3159395-supplementary.pdf]

Anti-Biofilm				
**Study Type**	**Alternative Compound**	**Title**	**Year**	**DOI**
Invitro	Extract of *Rubus idaeus* and *Rubus occidentalis*	Anti-Helicobacter pylori Biofilm Extracts from Rubus idaeus and Rubus occidentalis	2024	10.3390/pharmaceutics16040501
Invitro	Citropten, bergapten, and its positional isomer isolated from *Citrus sinensis L. l*eaves	Anti-Helicobacter pylori, anti-biofilm activity, and molecular docking study of citropten, bergapten, and its positional isomer isolated from Citrus sinensis L. leaves	2024	10.1016/j.heliyon.2024.e25232
Invitro	Aloe-emodin	Aloe-emodin destroys the biofilm of Helicobacter pylori by targeting the outer membrane protein 6	2024	10.1016/j.micres.2023.127539
Review	Turmeric and curcumin	Turmeric and curcumin as adjuncts in controlling Helicobacter pylori-associated diseases: a narrative review	2024	10.1093/lambio/ovae049
Invitro	Cinnamaldehyde of *Cinnamomum cassi*	Cinnamaldehyde: An effective component of Cinnamomum cassia inhibiting Helicobacter pylori	2024	10.1016/j.jep.2024.118222
Review	Antimicrobial peptides	Strategies adopted by gastric pathogen Helicobacter pylori for a mature biofilm formation: Antimicrobial peptides as a visionary treatment	2023	10.1016/j.micres.2023.127417
Invitro	Eugenol Clove Essential Oil	Antibacterial, Antibiofilm and Anti-Inflammatory Activities of Eugenol Clove Essential Oil against Resistant Helicobacter pylori	2023	10.3390/molecules28062448

**Anti-Biofilm**				
**Study Type**	**Alternative Compound**	**Title**	**Year**	**DOI**
Invitro	Bacillus and Enterococcus probiotic strains	The potential of Bacillus and Enterococcus probiotic strains to combat helicobacter pylori attachment to the biotic and abiotic surfaces	2023	10.1007/s10123-023-00347-z
Invitro	Carvacrol, Amoxicillin and Salicylhydroxamic Combination	Antimicrobial and Antibiofilm Activities of Carvacrol, Amoxicillin and Salicylhydroxamic Acid Alone and in Combination vs. Helicobacter pylori: Towards a New Multi-Targeted Therapy	2023	10.3390/ijms24054455
Invitro	*Acacia nilotica* Flower Extract	Pharmacological Evaluation of Acacia nilotica Flower Extract against Helicobacter pylori and Human Hepatocellular Carcinoma In Vitro and In Silico	2023	10.3390/jfb14040237
Invitro	Laurel Leaf Extract	Anti-Helicobacter pylori, Antioxidant, Antidiabetic, and Anti-Alzheimer's Activities of Laurel Leaf Extract Treated by Moist Heat and Molecular Docking of Its Flavonoid Constituent, Naringenin, against Acetylcholinesterase and Butyrylcholinesterase	2023	10.3390/life13071512
Invitro	demethylated hydroxylated phillygenin derivative	Therapeutic effect of demethylated hydroxylated phillygenin derivative on Helicobacter pylori infection	2023	10.3389/fmicb.2023.1071603
Invitro	Phillygenin	Phillygenin Inhibits Helicobacter pylori by Preventing Biofilm Formation and Inducing ATP Leakage	2022	10.3389/fmicb.2022.863624

Anti-Biofilm				
**Study Type**	**Alternative Compound**	**Title**	**Year**	**DOI**
Invitro	Arylaminoartemisinin GC012	In Vitro Activity of the Arylaminoartemisinin GC012 against Helicobacter pylori and Its Effects on Biofilm	2022	10.3390/pathogens11070740
Invitro	Moenomycin A	Combating multidrug-resistant Helicobacter pylori with moenomycin A in combination with clarithromycin or metronidazole	2022	10.3389/fchem.2022.897578
Invivo	M5N32	The Quinone-Derived Small Molecule M5N32 Is an Effective Anti-Helicobacter pylori Agent Both In Vivo and In Vitro	2022	10.1093/infdis/jiac401
Invitro	Lactobacillus plantarum LN66	In Vitro Effects of Lactobacillus plantarum LN66 and Antibiotics Used Alone or in Combination on Helicobacter pylori Mature Biofilm	2021	10.3390/microorganisms9020424
Invitro	Lactobacillus salivarius LN12	Effects of Lactobacillus salivarius LN12 in Combination with Amoxicillin and Clarithromycin on Helicobacter pylori Biofilm In Vitro	2021	10.3390/microorganisms9081611
Invitro	Carbonic Anhydrases by Carvacrol and Thymol	Selective Inhibition of Helicobacter pylori Carbonic Anhydrases by Carvacrol and Thymol Could Impair Biofilm Production and the Release of Outer Membrane Vesicles	2021	10.3390/ijms222111583
Invivo	Dihydrotanshinone I	Dihydrotanshinone I Is Effective against Drug-Resistant Helicobacter pylori In Vitro and In Vivo	2021	10.1128/AAC.01921-20

Anti-Biofilm				
**Study Type**	**Alternative Compound**	**Title**	**Year**	**DOI**
Invitro	*Chelidonium majus* and *Corydalis cheilanthifolia* Extracts	Antibiofilm and Antimicrobial-Enhancing Activity of Chelidonium majus and Corydalis cheilanthifolia Extracts against Multidrug-Resistant Helicobacter pylori	2021	10.3390/pathogens10081033
Clinical Trial	*Limosilactobacillus reuteri* Strains	Limosilactobacillus reuteri Strains as Adjuvants in the Management of Helicobacter pylori Infection	2021	10.3390/medicina57070733
Invitro	Dioscin, Hypericin, Amentoflavone, Aescin, etc	In Silico Screening and In Vitro Assessment of Natural Products with Anti-Virulence Activity against Helicobacter pylori	2021	10.3390/molecules27010020
Invitro	*Hibiscus rosa-sinensis L.* red flower	Antibacterial activity of Hibiscus rosa-sinensis L. red flower against antibiotic-resistant strains of Helicobacter pylori and identification of the flower constituents	2021	10.1590/1414-431X2020e10889
Invitro	Myricetin	Myricetin as an Antivirulence Compound Interfering with a Morphological Transformation into Coccoid Forms and Potentiating Activity of Antibiotics against Helicobacter pylori	2021	10.3390/ijms22052695

**OMV and Phage**		
**Study summary**	**Year**	**DOI**
S. typhimurium OMV having *Hp* antigens protects *Hp* Infection in mice	2024	10.1080/21505594.2024.2367783
HPy1r can inhibit *Hp* up to 24 hours	2022	10.3390/ijms23147885
Engineered endolysin and holin enzyme has bacteriostatic effect on *Hp*	2021	https://doi.org/10.1186/s13568-021-01222-8
Hp OMV as vaccine adjuvant have protective effect in mice	2020	10.3389/fmicb.2020.01340
Combination of Hpj phage-lactoferrin-hydroxyapatite have antimicrobial activity	2020	10.3390/microorganisms8081214
Hp OMV reduce infection via Th-2 in mice	2019	10.1093/femspd/ftz050
Hp OMV induce immunity in mice	2010	https://doi.org/10.1111/j.1462-5822.2009.01404.x

**Other Mechanisms**			
**Study type**	**Study summary**	**Year**	**DOI**
In vivo	Antimotillins inhibit stomach colonization	2022	10.1128/mbio.03755-21
In vitro	Moenomycin A, a cell wall transglycosilase inhibitor combats *Hp* MDR	2022	10.3389/fchem.2022.897578
In vivo	Optimized thienopyrimidines inhibit *Hp* via respiratory complex I	2021	10.1021/acsinfecdis.0c00300
In vitro	Analogue of *Hp* MTAN enzyme can inhibit the enzyme	2019	10.1021/acs.jmedchem.8b01642.
In vitro & in vivo	Flavodoxin inhibitors decrease gastric colonization and eradicate infection up to 60%	2019	10.1021/acs.jmedchem.9b00355
In vitro & in vivo	Vitamin D3 drives *Hp* elimination via atolysosomal pathway	2019	10.1080/15548627.2018.1557835
In vitro & In vivo	Agonist of TRPML-1 reverse VacA endolysosomal trafficking	2019	10.1038/s41564-019-0441-6.
In vitro	Polysaccharide sulfate inhibit *Hp* adhesion	2014	10.1016/j.carbpol.2013.12.063

**Probiotics**			
**Study type**	**Study summary**	**Year**	**DOI**
In vivo	*L. plantarum* ZFM4 reduce gastric inflammatory response and inhibit *Hp* growth	2024	10.3389/fcimb.2023.1320819
In vivo	*L. plantarum* Q21, Q45, and Q85 from Chinese fermented food inhibit *Hp* and improve symptoms	2024	10.3390/foods13121851
In vitro	*Lactobacillus gasseri* BIO6369 and *Lacticaseibacillus rhamnosus* BIO5326 decreased epithelial-mesenchymal transition	2024	10.1111/hel.13108.
In vitro	Lactobacilli affects *Hp* motility, acid tolerance, and AMP survival	2022	10.3390/ijms232415451
RCT	*Saccharomyces boulardii* partially cures *Hp* infection before bismuth quadruple therapy	2022	10.3389/fcimb.2022.903002.
RCT	Combination of *L. acidophilus, L. plantarum, B. lactis* and *S. boulardii* and non BQT had higher *Hp* eradication and improve symptoms	2022	10.3390/nu14030632
RCT	A combination of Bifidobacterium infantis , Lactobacillus acidophilus, Enterococcus faecalis, and Bacillus cereus and BQT had no significant eradication rate but improve GI symtomps	2022	10.3389/fimmu.2022.1033063
In vitro	*L. casei* T1 and its supernatant had potent inhibiory effect on *Hp* growth	2021	10.1016/j.jff.2021.104611
In vivo	Lactobacillus fermentum P2 (P2), L. casei L21 (L21), L. rhamnosus JB3 (JB3) reduce gastric inflammation in mice	2020	10.3390/nu12082476
Clinical Trial	*C. butyricum* and *B. coagulans* inhibit *Hp* to some extent with rare side effects	2020	10.1097/MD.0000000000022976

**Probiotics**			
**Study type**	**Study summary**	**Year**	**DOI**
In vitro	*L. casei, L. paracasei* and *L. acidophilus* can inhibit 57 *Hp* strains	2020	10.3390/antibiotics9050244
In vitro	*Lactobacillus plantarum* ZDY2013 suppress growth and urease acivity of *Hp*	2018	10.1016/j.jbiosc.2018.04.003
In vivo	*L. fermentum* UCO-979C inhibit *Hp* strain SS1 in mongolian gerbil	2018	10.3920/BM2017.0160
RCT	*C. butyricum* supplementation imptove GI symptoms in patients	2018	10.1016/j.ebiom.2018.08.028
RCT	Probiotics supplementation (*E. faecium and B. subtilis)* helps construct gut microbiome after eradication therapy	2018	10.1007/s40121-020-00372-9.

Nanoparticles			
**Study type**	**Study summary**	**Year**	**DOI**
In vivo	Chitosan microspheres bind *Hp* and reduce infection in mice	2020	10.1016/j.actbio.2020.06.035.
In vitro	LMW Dextran sulfate nanocapsules inhibit *Hp* adhesion	2019	10.1021/acsabm.9b00523
In vitro	*Hp*-mimic nanoparticles reduces *Hp* adhesion in AGS cells	2019	10.1002/anie.201906280
In vitro	AGS-NPs loaded with clarithromycin has superior theraprutic efficacy than regular clarithromycin	2019	10.1002/adtp.201800016
In vitro & in vivo	UreI-mediated amoxicillin-PLGA/ureido-conjugated chitosan nanoparticles have better anti *Hp* activity compared to regular amoxicillin	2018	10.1016/j.colsurfb.2018.01.008
In vitro & in vivo	A graphitic nanozyme had bactericidal activity towards *Hp*	2021	10.1038/s41467-021-22286-x
In vitro	Delivery of oligonucleotides by fusogenic liposomesthrough mucus to *Hp*	2017	10.1016/j.biomaterials.2017.05.029
In vitro & in vivo	Sonodynamic therapy mediated by lecithin bilayer-coated poly nanoparticle loaded with verteporfin inactivates *Hp* by generating ROS	2024	10.1038/s41467-024-45156-8
In vitro	Silver Ultra-NanoClusters (SUNCs) can eradicate mature biofilm and had low toxicity on human cells	2020	10.3389/fmicb.2020.01705
In vitro & in vivo	pH responsive metal-organic framework hydrogen-generation nanoparticle kills *Hp*, alleviate inflammation, and restore impaired gastric mucosa	2022	10.1002/adma.202105738
